# In Vitro Effect of Using Endo‐Activator on Pushout Bond Strength of Radicular Dentin to Prefabricated Fiber Post in Using Natural Matrix Metalloproteinase Inhibitors

**DOI:** 10.1002/cre2.70255

**Published:** 2025-11-28

**Authors:** Nadia Elyassi Gorji, Homayoun Alaghemand, Faraneh Mokhtarpour, Elham Mahmodnia

**Affiliations:** ^1^ Department of Operative Dentistry, Faculty of Dentistry, Esthetic and Restorative Dentistry Babol University of Medical Sciences Babol Iran; ^2^ Department of Operative Dentistry, Dental Materials Research Center, Dental School Babol University of Medical Sciences Babol Iran; ^3^ Restorative Dentistry Specialist Babol Iran; ^4^ Department of Endodontic, Faculty of Dentistry Babol University of Medical Sciences Babol Iran

**Keywords:** adhesives, aloe, dental pulp cavity, matrix metalloproteinases

## Abstract

**Objectives:**

This study assessed the effect of using Endo‐Activator on pushout bond strength (PBS) of radicular dentin to prefabricated fiber post in using epigallocatechin gallate (EGCG) and aloe vera natural matrix metalloproteinase (MMP) inhibitors.

**Materials and Methods:**

This in vitro study was conducted on 120 extracted teeth in 2 groups with/without using Endo‐Activator for root canal irrigation, each with 3 subgroups of LuxaBond primer (I) alone, (II) with aloe vera, and (III) with EGCG. After root canal treatment and post space preparation, radicular dentin was etched, the respective primer was rubbed on radicular dentin, and fiber posts were cemented using LuxaBond dual‐cure adhesive and LuxaCore Z dual‐cure cement. Each tooth was horizontally sectioned at the coronal, middle, and apical thirds, and the PBS of fiber post to dentin was measured immediately and after 6 months. Failure mode was determined under a stereomicroscope (×80).

**Results:**

Irrigation with Endo‐Activator improved both the immediate and 6‐month PBS in all sections (*p* < 0.05). The highest immediate PBS was recorded in the coronal third in the aloe vera subgroups with no significant difference between use/no use of Endo‐Activator (*p* > 0.05). At 6 months, aloe vera yielded the highest PBS in the coronal third, with significantly higher values in use of Endo‐Activator (*p* < 0.05).

**Conclusion:**

Irrigation with Endo‐Activator along with the application of EGCG and aloe vera significantly improved the PBS of fiber post to radicular dentin and its durability; this effect was greater in the cervical third in the aloe vera subgroups both immediately and after 6 months.

**Abbreviations**
DC: dual‐cure
EGCG: epigallocatechin gallate
MMP: matrix metalloproteinases
PBS: push‐out bond strength
PSP: post space preparation
RCT: root canal treatment

## 1 Introduction

Fiber‐reinforced intracanal posts are commonly used for restoration of endodontically treated teeth due to provision of sufficient retention and optimal esthetics, and having a modulus of elasticity comparable to that of dentin (Ghazvehi et al. 2022). They provide retention for the core, and transfer the applied loads to the roots to protect the tooth against root fracture (Ghazvehi et al. 2022). Nonetheless, achieving a strong bond between the resin cement and radicular dentin is challenging, and this bond may also undergo degradation over time (Ghazvehi et al. 2022). However, prefabricated posts are still recommended since they can bond to radicular dentin. A number of factors such as the cementation technique, type of post, adaptation to post space, dentin pretreatment, and procedural steps can affect the retention of fiber posts in the root canal system. Therefore, it is imperative to achieve optimal adhesion between the radicular dentin, resin cement, and fiber post. It is noteworthy that the dentin‐resin bond strength may be compromised by degradation of the adhesive system components following water sorption and/or proteolytic degradation of the hybrid layer due to the action of matrix metalloproteinases (MMPs) activated by the cysteine cathepsins usually available in the form of a zymogen (Victorino et al. 2016). Zymogens are activated when the teeth are stimulated by thermal alterations, acidic erosion, caries, or mechanical preparation to dissolve the dentin collagen matrix. In this process, the unprotected collagen fibers of the hybrid layer are susceptible to degradation and breakdown, and destruction of collagen would degrade the hybrid layer and subsequently decrease the bond strength. Thus, application of MMP inhibitors in the bonding process may prevent the degradation of collagen fibers and improve the resin‐dentin bond strength and durability (Zhao et al. 2023).

Since dentin has organic (collagen‐rich extracellular matrix) and mineral (calcium apatite crystals) components, bonding to dentin has always been challenging (de Paris Matos et al. 2020). Many attempts have been made to improve the quality and longevity of resin‐dentin bond by increasing the collagen resistance against enzymatic degradation. Dentin bio‐modifiers such as endogenous/exogenous MMP inhibitors, and collagen crosslinkers are used for preparation of demineralized dentin, or are incorporated in the composition of bonding agents (Mazzoni et al. 2018). Application of natural crosslinkers with optimal biocompatibility, insignificant cytotoxicity, and medium reaction speed has recently gained the attention of researchers. Crosslinkers can transversely bond to proteases and interfere with their molecular mobility. They can also inactivate the C‐terminal telopeptides and subsequently prevent the attachment of collagenase to peptide bonds and protect the C‐terminal telopeptides. Thus, it is important to compare different crosslinkers to find the most efficient types for clinical use (Hass et al. 2016).

Epigallocatechin gallate (EGCG) is a catechin with the highest percentage in green tea extract (49%), which can serve as a natural MMP inhibitor (Ismiyatin et al. 2022). It has high affinity to metal ions and can inhibit the activity of MMPs through chelation and improve the integrity and stability of collagen. Thus, it can increase the hybrid layer durability and adhesion (Ismiyatin et al. 2022). Evidence shows that EGCG can serve as a crosslinker, and form hydrogen bonds to collagen peptide chain. It can replace the water molecules in the collagen bonding chain and decrease the interactions of collagen with water, making it more hydrophobic. Resultantly, the monomer can better penetrate into the collagen fibers and prevent water sorption, improving the monomer‐dentin bond strength (Ismiyatin et al. 2022).

Aloe vera is another natural MMP inhibitor known for its optimal antioxidant, anti‐inflammatory, and antimicrobial properties. Aloe vera gel has inhibitory effects on MMP‐2 and MMP‐9, and is highly effective for dentin collagen stabilization, preventing its degradation (Moustafa et al. 2020).

It should be noted that in addition to the above, other factors such as the quality of cleaning of dentinal tubules and the quality and quantity of cleaning of secondary canals in interradicular areas can also affect the adhesion and durability of bonding; therefore, using a process that can improve this can also affect the bond strength. One of the methods used for this purpose during root canal treatment is the use of Endo‐Activator, which has not been previously evaluated in restorative dentistry. Endo‐Activator is a sonic irrigation activator system, designed to agitate the intracanal irrigating solutions. It has shown superior irrigation efficacy compared with the conventional syringe irrigation (de Gregorio et al. 2009). It is composed of a portable handpiece and three disposable flexible polymer tips in variable sizes that do not cut through the dentin (Mancini et al. 2013). Endo‐Activator can optimally clean the root canal system, accessory canals, fins, and apical deltas by energizing the intracanal irrigants through its non‐cutting flexible polymer tips (Ruddle 2015). Also, it can improve sealer penetration into radicular dentin (Bolles et al. 2013). However, its effect on the pushout bond strength (PBS) of fiber post to radicular dentin is a matter of question.

Considering the widespread use of fiber posts, the aforementioned problems of bonding to dentin, and scarcity of studies on natural MMP inhibitors, this study aimed to assess the effect of Endo‐Activator on PBS of prefabricated fiber post to root dentin in using EGCG and aloe vera as natural MMP inhibitors. The null hypothesis of the study was that simultaneous use of Endo‐Activator and EGCG or aloe vera would not significantly increase the PBS.

## 2 Materials and Methods

This in vitro, experimental study was conducted on single‐rooted single‐canal human teeth extracted for purposes not related to this study (such as orthodontic treatment or hopeless periodontal prognosis). The attached tissue residues were removed, and the teeth were stored in a screw‐top container containing 0.04% thymol. The study protocol was approved by the ethics committee of Babol University of Medical Sciences (IR.MUBABOL.HRI.REC.1402.082).

### 2.1 Sample Size

The sample size was calculated to be 20 in each group (a total of 120 in 6 groups) according to a previous study (Sorourhomayoun et al. 2021) and assuming *α *= 0.05 and *β *= 0.2.

### 2.2 Eligibility Criteria

The inclusion criteria were freshly extracted single‐rooted single‐canal teeth with mature apices and almost equal root length (Jouhar 2021).

The exclusion criteria were teeth with any type of root or root canal curvature in any direction (mesial, distal, buccal, or lingual) as detected on radiographs taken during endodontic treatment, fracture lines, internal/external root resorption, or root caries (Jouhar 2021).

### 2.3 Specimen Preparation

Eligible teeth were decoronated by a cutting disc (D&Z, Drendel + Zwilling, Berlin, Germany) and low‐speed handpiece. The roots then underwent root canal treatment. The root canals were negotiated by a stainless‐steel hand K‐file (Dentsply Maillefer, Ballaigues, Switzerland) and filed to #80 using the step‐back technique after working length determination. Root canal irrigation was performed with sodium hypochlorite (Niclor 5; Ogna, Muggiò, Italy) and saline (#40 master apical file). The root canals were subsequently obturated with gutta‐percha (AriaDent, Asia Chemi Teb, Tehran, Iran) and AH26 sealer (Dentsply Sirona, Konstanz, Germany) using the vertical condensation technique. Optimal quality of obturation was confirmed radiographically. The coronal part of the root canals was then sealed with light‐cure glass‐ionomer cement (Fuji VII, GC Corp., Tokyo, Japan). Next, the roots were immersed in distilled water for 1 week in order for the sealer to completely set (Ghazvehi et al. 2022). The post space was subsequently prepared after removal of glass ionomer from the coronal part of the root using #3 peeso reamer (Dentsply Sirona, Konstanz, Germany) such that a minimum of 4 mm of gutta‐percha remained in the root canal (Bengoa et al. 2020).

### 2.4 Intra‐Radicular Dentin Treatment

After post space irrigation with distilled water and drying with #60 paper points (Meta BioMed, Seoul, South Korea), intra‐radicular dentin was etched with 37% phosphoric acid (Etching Gel, DMG, Hamburg, Germany) for 15 s, and rinsed. A microbrush was also used for better removal of the etching gel. Excess moisture was eliminated by using a #60 paper point. The roots were then randomly assigned to 2 groups (*n* = 60) for irrigation with or without Endo‐Activator sonic irrigation device (Dentsply Sirona, Konstanz, Germany). After etching the canal and cleaning the remaining etchant using a microbrush, the entire canal length was irrigated with saline 3 times to remove coarse debris; then, the blue head of the device (large size) was selected based on the size and length of the canal (which was matched with the same drill in all samples) in such a way that the tip of the head easily reached the end of the canal length (without pressing on the walls). In the next step, the entire canal length was filled with sterile water using a 30‐gauge needle, and the vibration frequency was applied for 10 s per tooth. The entire canal length was rinsed again with sterile water 3 times to completely remove fine debris.

Each group was subsequently divided into the following 3 subgroups (*n* = 20):

Control subgroup: Only the pre‐bond (LuxaBond; DMG, Hamburg, Germany) was used in this subgroup.

EGCG subgroup: EGCG extract powder (Giahkala, Tehran, Iran) in 200 μg/mL concentration (Bengoa et al. 2020) was added to the pre‐bond.

Aloe vera subgroup: Aloe vera (Giahkala, Tehran, Iran) with 99% purity in 2 mg/100 mL concentration (Moustafa et al. 2020) was added to the pre‐bond.

All materials were prepared and used fresh to ensure their maximum efficacy. In each subgroup, the respective pre‐bond was rubbed on the radicular dentin surface for 20 s, and excess material was removed by a #60 paper point. After primer solvent evaporation by gentle oil‐free air spray, equal amounts of components A and B of LuxaBond bonding agent (DMG, Hamburg, Germany) were mixed and rubbed on radicular dentin in the post space for 30 s. A paper point was used to remove excess material, and the solvent was evaporated by gentle oil‐free air spray.

### 2.5 Fiber Post Cementation

In the next step, #3 fiber posts (FGM, Joinville, Brazil) were cleaned with 96% ethanol (Taghtir Khorasan, Shimaz, Khorasan, Iran) and silanated (Bisco silane, Bisco Inc., Schaumburg, USA). Dual‐cure cement (LuxaCore Z, DMG, Schaumburg, Germany) was injected into the root canal space, and the fiber post was placed in the prepared post space with several pull and push movements to ensure its complete adaptation and better penetration of cement into dentin irregularities. Light polymerization was performed using a light‐curing unit (BluePhase C8, Ivoclar Vivadent, NY, USA) with a light intensity of 800 mW/cm^2^ for 20 s. Excess cement was removed (Table 1).

**Table 1:** Details of fiber post surface pretreatments, chemical compositions, batch numbers, and application modes of materials used in this study.

Material	Composition	Application mode
Luxacore Z DC cement, DMG (LOT 290892)	Bis‐GMA, UDMA, Barium glass, colloidal silica, nanocomposite, zirconium dioxide 71% weight	Dispense the cement in the post space and insert the post.
DMG etching gel	37% orthophosphoric acid gel.	Etch tooth structure for 15 s.
LuxaBond DC, DMG (LOT 289060)	Pre‐bond: Ethanolic aryl sulfonate solution. Bond A: Resin matrix (HEMA, bis‐GMA, MDP) approx. 97%, catalyst, stabilizer, additives. Bond B: Ethanol approx. 57%, water approx. 35%, catalyst (benzoyl peroxide)	Use a brush to work 1 to 2 drops of pre‐bond into the etched tooth structure. Remove any excess, e.g., with a paper tip. Blow gently using oil and water‐free air. The surface should appear evenly moist. Mix one drops each of bond A and bond B in a mixing pad in a 1:1 ratio for approx. 0.5 s. Apply a layer of the bond mix on to the preparation using a micro brush and work into the tooth structure for 20 s. Direct a stream of air at the material for 10 s. Apply another layer of the bond mix onto the preparation using a micro brush and work into the tooth structure for 20 s. Direct a stream of air at the material for 10 s.
EGCG extract powder, Giahkala (Batch no: Pe10021204)	Pure extract powder of epigallocatechin‐3‐gallate	200 µg/mL of powdered extract was added to pre‐bond.
Aloe vera extract powder, Giahkala (Batch no: Pe10021276)	Pure extract powder of aloe vera	2 mg of aloe vera powder with 99% purity per 100 mL was added to pre‐bond.

### 2.6 Preparation of Root Sections

**Figure 1:**
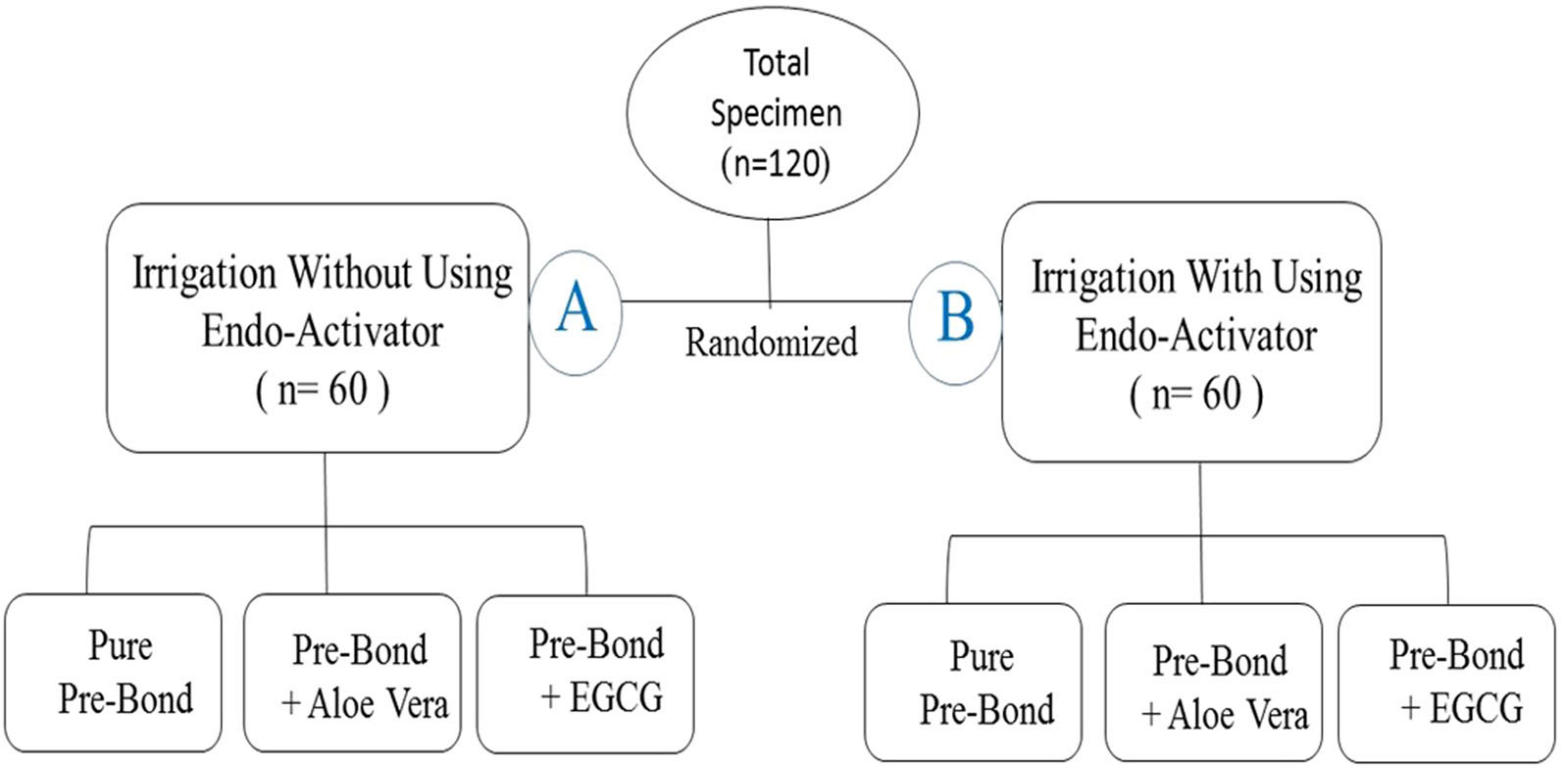
Classification of study samples.

In each group, the roots were fixed in epoxy resin, and mounted on the pad of the sectioning machine (Precision Sectioning Machine D; Nemo, USA) perpendicular to their longitudinal axis using cyanoacrylate glue. A 4‐inch low‐speed diamond saw (Microremet, Remet, Bologna, Italy) was then used to section slices with 1 mm thickness from the cervical, middle, and apical thirds of the post space under water coolant. The cervical third section was made at least 2 mm below the cementoenamel junction. The middle third section was made at least 5 mm below the cementoenamel junction, and the apical third section was made at the apical 2 mm of the fiber post‐radicular dentin contact area; accordingly, the slices were obtained from the most central parts of each root third (Figure 1).

### 2.7 Measuring the PBS

The diameter and thickness of the slices were measured by a digital caliper (Mitutoyo, Tokyo, Japan). Half of the specimens in each subgroup (*n* = 10) were transferred to a universal testing machine (Koopa TB‐5T, Mashhad, Iran) and subjected to the application of progressively increasing pushout load towards the apical at a crosshead speed of 1.0 mm/minute until fracture to determine the PBS of fiber post‐dentin (Bengoa et al. 2020). To eliminate the effect of Poisson's ratio, the cross‐section of the piston was fully adapted to the fiber post periphery. To do so, the periphery of the fiber post embedded in resin cement was outlined by a marker. The PBS values obtained in Newtons (N) were divided by the root canal surface area (mm^2^) to determine the PBS in megapascals (MPa). The root canal surface area was calculated using the following formula:

A=P×h

where *A* is the root canal surface area in square millimeters (mm^2^), *P* is the root canal periphery in millimeters (mm), and *h* is the dentinal section height in millimeters (mm) (Bengoa et al. 2020). The obtained values were recorded as the immediate PBS of the specimens. The remaining teeth in each subgroup were stored in distilled water at 37°C for 6 months and then underwent PBS testing as mentioned above to assess the PBS of the specimens in the long‐term (Du et al. 2012).

### 2.8 Failure Mode

The failure mode of the specimens was determined by their inspection under a stereomicroscope (SMZ 1000, Nikon, Minato City, Tokyo, Japan) at ×80 magnification as follows, and reported in percentage:

– Adhesive failure at the resin cement–radicular dentin interface
– Adhesive failure at the resin cement–fiber post interface
– Cohesive failure within the cement
– Mixed failure

### 2.9 Statistical Analysis

Considering the non‐normal distribution of data as shown by the Kolmogorov–Smirnov test, the Wilcoxon test was applied to compare the immediate and 6‐month PBS of the subgroups. The Kruskal–Wallis test was used for within‐group comparison of PBS with/without using Endo‐Activator. The PBS was compared among the cervical, middle, and apical thirds by the Kruskal–Wallis test. Pairwise comparisons were carried out by the Dunn–Bonferroni test. ANCOVA was used to compare the 6‐month PBS after controlling for the effect of immediate PBS. All statistical analyses were performed using SPSS version 20 (SPSS Inc., IL, USA) at 0.05 level of significance.

## 3 Results

### 3.1 Between‐Group Comparison of PBS

As shown in Table 2, a significant difference was found in PBS in all sections among the six subgroups both immediately and 6 months after the intervention (*p* < 0.05). The mean PBS significantly decreased after 6 months only in the apical third of the Endo‐Activator/control subgroup (*p* < 0.05). Pairwise comparisons are presented in Table 3.

**Table 2:** Mean PBS (MPa) of the six subgroups immediately and 6 months after the intervention in the cervical, middle, and apical thirds.

Section	Subgroup	Time		*p*‐value
		Immediately	6 months	
Cervical	No Endo‐Activator control	26.50 ± 14.55	22.70 ± 6.96	0.83
	No Endo‐Activator EGCG	29.90 ± 8.04	31.60 ± 8.83	0.47
	No Endo‐Activator aloe vera	35.60 ± 9.87	32.40 ± 6.38	0.76
	Endo‐Activator control	32.80 ± 10.79	30.30 ± 7.07	0.33
	Endo‐Activator EGCG	37.30 ± 7.78	37.00 ± 8.69	0.61
	Endo‐Activator aloe vera	46.80 ± 16.06	43.30 ± 9.21	0.55
*p*‐value		0.004*	< 0.001*	—
Middle	No Endo‐Activator control	25.20 ± 14.19	19.10 ± 6.43	0.35
	No Endo‐Activator EGCG	27.40 ± 8.16	27.30 ± 11.10	0.99
	No Endo‐Activator aloe vera	29.60 ± 5.66	26.90 ± 6.62	0.38
	Endo‐Activator control	31.20 ± 6.71	26.20 ± 7.88	0.23
	Endo‐Activator EGCG	37.30 ± 12.97	33.80 ± 7.09	0.44
	Endo‐Activator aloe vera	37.00 ± 11.10	33.50 ± 9.80	0.33
*p*‐value		0.04*	0.001*	—
Apical	No Endo‐Activator control	20.40 ± 11.08	18.70 ± 7.10	0.90
	No Endo‐Activator EGCG	22.90 ± 6.93	22.00 ± 8.66	0.50
	No Endo‐Activator aloe vera	23.50 ± 6.65	21.10 ± 6.50	0.44
	Endo‐Activator control	30.60 ± 7.02	22.50 ± 7.84	0.01*
	Endo‐Activator EGCG	32.70 ± 5.51	29.70 ± 8.95	0.34
	Endo‐Activator aloe vera	32.10 ± 7.20	31.10 ± 6.38	0.64
*p*‐value		0.001*	0.005*	—

* Significant at 0.05; values are reported as mean ± standard deviation.

**Table 3:** Pairwise comparison of the subgroups regarding immediate and 6‐month PBS (MPa) by the Dunn–Bonferroni test.

Group (I)	Group (J)	Cervical		Middle		Apical	
		Immediate	6 months	Immediate	6 months	Immediate	6 months
No Endo‐Activator control	No Endo‐Activator EGCG	0.31	0.07	0.54	0.08	0.42	0.40
	No Endo‐Activator aloe vera	0.03*	0.02*	0.19	0.04*	0.27	0.50
	Endo‐Activator control	0.12	0.10	0.08	0.07	0.005*	0.32
	Endo‐Activator EGCG	0.008*	0.001*	0.01*	< 0.001*	0.001*	0.006*
	Endo‐Activator aloe vera	< 0.001*	< 0.001*	0.007*	< 0.001*	0.002*	0.001*
No Endo‐Activator EGCG	No Endo‐Activator aloe vera	0.25	0.71	0.48	0.78	0.76	0.86
	Endo‐Activator control	0.58	0.85	0.25	0.96	0.04*	0.87
	Endo‐Activator EGCG	0.09	0.14	0.04*	0.02*	0.008*	0.04*
	Endo‐Activator aloe vera	0.005*	0.006*	0.03*	0.07	0.01*	0.01*
No Endo‐Activator aloe vera	Endo‐Activator control	0.55	0.58	0.65	0.81	0.09	0.74
	Endo‐Activator EGCG	0.59	0.27	0.21	0.04*	0.01*	0.03*
	Endo‐Activator aloe vera	0.09	0.01*	0.17	0.13	0.03*	0.008*
Endo‐Activator control	Endo‐Activator EGCG	0.26	0.09	0.43	0.02*	0.50	0.07
	Endo‐Activator aloe vera	0.02*	0.003*	0.35	0.08	0.70	0.02*
Endo‐Activator EGCG	Endo‐Activator aloe vera	0.25	0.20	0.88	0.66	0.76	0.58

* Significant at 0.05.

### 3.2 Within‐Group Comparison of PBS Over a 6‐Month Period

As shown in Table 4, a significant reduction occurred in the mean PBS only in the Endo‐Activator/control subgroup in the apical third (*p* = 0.01).

**Table 4:** Within‐group comparison of PBS (MPa) in the cervical, middle, and apical thirds over a 6‐month period.

Group	Section	Time		*p*‐value
		Immediately	6 months	
No Endo‐Activator/control	Cervical	25.50 ± 14.55	22.70 ± 6.96	0.83
	Middle	25.20 ± 14.19	19.10 ± 6.43	0.35
	Apical	20.40 ± 11.08	18.70 ± 7.10	0.90
*p*‐value		0.33	0.29	
No Endo‐Activator/EGCG	Cervical	29.90 ± 8.04	31.60 ± 8.83	0.47
	Middle	27.40 ± 8.16	27.30 ± 11.10	0.99
	Apical	22.90 ± 6.93	22.00 ± 8.66	0.50
*p*‐value		0.12	0.07	—
No Endo‐Activator/aloe vera	Cervical	35.60 ± 9.87	32.40 ± 6.38	0.76
	Middle	29.60 ± 5.66	26.90 ± 6.62	0.38
	Apical	23.50 ± 6.65	21.10 ± 6.50	0.44
*p*‐value		0.01*	0.005*	—
Endo‐Activator/control	Cervical	32.80 ± 10.79	30.30 ± 7.07	0.33
	Middle	31.20 ± 6.71	26.20 ± 7.88	0.23
	Apical	30.60 ± 7.02	22.50 ± 7.84	0.01*
*p*‐value		0.97	0.11	—
Endo‐Activator/EGCG	Cervical	37.30 ± 7.78	37.00 ± 8.69	0.61
	Middle	37.30 ± 19.97	33.80 ± 7.09	0.44
	Apical	32.70 ± 5.51	29.70 ± 8.95	0.34
*p*‐value		0.48	0.09	—
Endo‐Activator/aloe vera	Cervical	46.80 ± 16.06	43.30 ± 9.21	0.55
	Middle	37.00 ± 11.10	33.50 ± 9.80	0.33
	Apical	32.10 ± 7.20	31.10 ± 6.38	0.64
*p*‐value		0.04*	0.008*	—

* Significant at 0.05; values are reported as mean ± standard deviation.

As shown in Table 4, a significant difference was noted in the mean immediate (*p* < 0.05) and 6‐month (*p* < 0.05) PBS among the three root sections in both the aloe vera subgroups with and without Endo‐Activator. Pairwise comparisons of the sections regarding the mean PBS in the no Endo‐Activator aloe vera subgroup revealed that the mean immediate PBS in the apical third was significantly lower than that in the cervical (*p* = 0.003) and middle (*p* = 0.04) thirds. The 6‐month PBS in the apical third was significantly lower than that in the cervical third (*p* = 0.001).

Pairwise comparisons of the sections regarding the mean PBS in the Endo‐Activator aloe vera subgroup revealed that the mean immediate PBS in the apical third was significantly lower than that in the cervical third (*p* = 0.02). The 6‐month PBS in the cervical third was significantly higher than that in the apical third (*p* = 0.004) and higher than that in the middle third (*p* = 0.01).

### 3.3 Comparison of the 6‐Month PBS After Controlling for the Effect of Immediate PBS

The mean 6‐month PBS in the no Endo‐Activator/control subgroup was significantly lower than that in the Endo‐Activator/aloe vera subgroup by 15.52 units. The mean 6‐month PBS in the no Endo‐Activator/EGCG, no Endo‐Activator/aloe vera, and Endo‐Activator/control subgroups was significantly lower than that in the Endo‐Activator/aloe vera subgroup by an average of 8.77, 8.99, and 9.49 units, respectively. The mean 6‐month PBS was also significantly different among different sections (*p* < 0.05), such that by controlling for the effect of immediate PBS, the mean 6‐month PBS in the cervical and middle thirds increased by an average of 8.55 and 3.52 units, respectively, compared to the apical third.

### 3.4 Failure Mode

As shown in Table 5, no cohesive failure occurred. The majority of fractures immediately after the intervention and after 6 months were adhesive at the dentin‐cement interface, and mixed. Dentin‐cement adhesive failure was more common in the apical third while mixed failure was more frequent in the cervical third.

**Table 5:** Frequency of different failure modes.

	Immediately				After 6 Month		
	Failure‐type root section	Dentin‐resin	Fiber‐ resin	Mixed	Dentin‐ resin	Fiber‐ resin	Mixed
**Endo‐Activator‐control**	Cervical	4 (40%)	1 (10%)	5 (50%)	4 (40%)	2 (20%)	4 (40%)
	Medial	4 (40%)	0	6 (60%)	5 (50%)	1 (10%)	4 (40%)
	Apical	6 (60%)	0	4 (40%)	7 (70%)	0	3 (30%)
**Endo‐Activator ‐EGCG**	Cervical	5 (50%)	0	5 (50%)	2 (20%)	1 (10%)	7 (70%)
	Medial	6 (60%)	2 (20%)	2 (20%)	8 (80%)	2 (20%)	0
	Apical	7 (70%)	0	3 (30%)	6 (60%)	0	4 (40%)
**Endo‐Activator‐aloe vera**	Cervical	3 (30%)	2 (20%)	5 (50%)	4 (40%)	1 (10%)	5 (50%)
	Medial	2 (20%)	2 (20%)	6 (60%)	3 (30%)	2 (20%)	5 (50%)
	Apical	4 (40%)	0	6 (60%)	5 (50%)	1 (10%)	4 (40%)
**No Endo‐Activator ‐control**	Cervical	9 (90%)	0	1 (10%)	6 (60%)	0	4 (40%)
	Medial	5 (50%)	1 (10%)	4 (40%)	6 (60%)	0	4 (40%)
	Apical	8 (80%)	0	2 (20%)	7 (70%)	0	3 (30%)
**No Endo‐Activator ‐EGCG**	Cervical	5 (50%)	1 (10%)	4 (40%)	7 (70%)	0	3 (30%)
	Medial	4 (40%)	0	6 (60%)	3 (30%)	1 (10%)	6 (60%)
	Apical	6 (60%)	1 (10%)	3 (30%)	2 (20%)	1 (10%)	7 (70%)
**No Endo‐Activator ‐aloe vera**	Cervical	1 (10%)	3 (30%)	6 (60%)	1 (10%)	1 (10%)	8 (80%)
	Medial	5 (50%)	0	5 (50%)	4 (40%)	0	6 (60%)
	Apical	6 (60%)	1 (10%)	3 (30%)	3 (30%)	0	7 (70%)

*Note:* Cohesive failure alone did not occur in any specimen.

## 4 Discussion

The results showed that simultaneous use of Endo‐Activator and EGCG or aloe vera significantly increased the PBS of fiber post to radicular dentin and its durability. Thus, the null hypothesis of the study was rejected. However, application of EGCG or aloe vera alone and without using Endo‐Activator only caused a significant improvement in bonding durability in the aloe vera subgroup.

Since LuxaBond is a fourth generation (gold standard) dual‐cure bonding agent and has the same manufacturer as LuxaCore core build‐up composite, it was considered the most compatible for bonding and cement adhesion, and was therefore used in this study.

In line with the present results, it has been reported that aloe vera can inhibit MMP‐2 and MMP‐9 (Kudalkar et al. 2014). Also, (Bhandari et al. 2021). indicated that aloe vera has the potential to inhibit MMPs in human dentin collagen with/without dentin bonding agents. Previous studies also showed the inhibitory effect of EGCG on MMPs (especially MMP‐9) (Vidal et al. 2014) and indicated its positive effect on bond strength to root dentin (Ismiyatin et al. 2022; de Macedo et al. 2019; Garcia‐Contreras et al. 2023; Messias et al. 2021; Pheenithicharoenkul and Panichuttra 2016). However, no previous study has compared the effects of aloe vera and EGCG and their activation on bond strength.

Presence of the hybrid layer is imperative for bonding to root dentin. Two methods have been proposed to preserve the hybrid layer: (I) inhibition of enzymatic activity (especially MMPs), and (II) increasing the collagen resistance to degradation. MMP‐2 followed by MMP‐9 are the most abundant MMPs in human dentin, and play a critical role in collagen degradation. Increased activity of MMP‐2 and MMP following adhesive application, irrespective of the adhesive system, has been reported. Thus, the mechanism of action of MMP inhibitors is explained by their effect on both of the abovementioned methods for hybrid layer preservation (Breschi et al. 2018). On the other hand, cross‐linking of the dentin collagen matrix is a natural mechanism in dentin that creates tensile strength; application of enzyme inhibitors creates an enzyme‐substrate complex that prevents collagen hydrolysis, and can increase the bond strength (Thakur et al. 2022).

The present results showed that aloe vera significantly increased the PBS both with and without Endo‐Activator while EGCG significantly increased the PBS only when used in combination with Endo‐Activator. EGCG enhances the differentiation of odontoblast‐like cells and improves adhesion to dentin. It also improves hydrophobicity of dentin substrate and thermal stability of dentin collagen, decreasing nano‐leakage and improving resin‐dentin bonding durability (Garcia‐Contreras et al. 2023; Bryce et al. 2018). High penetration depth of aloe vera into dentinal tubules was previously reported, suggesting it as an alternative to chlorhexidine for dentin surface disinfection (Bhandari et al. 2021). Additionally, aloe vera contains aloin, which can change the concentration of calcium ions required for enzymatic activity. Furthermore, aloins have structural similarity to tetracycline, which is known for its MMP inhibitory effect (Aziz and Al Zaka 2019; Bitter et al. 2006).

(Thakur et al. 2022) reported that using Endo‐Activator significantly increased the efficiency of root canal debridement, which supports the significant increase in PBS in Endo‐Activator/EGCG and Endo‐Activator/aloe vera subgroups observed in the current study. (Bryce et al. 2018). confirmed the superior efficacy of Endo‐Activator to conventional syringe irrigation for removal of intracanal collagen film due to its better penetration into the dentinal tubules, and more effective elimination of smear layer and bacterial biofilm. (Aziz and Al Zaka 2019) compared the efficacy of XP‐Endo Finisher, XP‐Endo Finisher R, Canal Brush, and Endo‐Activator for elimination of calcium hydroxide from the root canal system. They found a significant difference in the cleaning efficiency of Endo‐Activator in the apical, middle, and coronal thirds such that it had a lower efficiency in the apical third than the cervical third. Also, Endo‐Activator group showed the highest calcium hydroxide residues in the middle and apical thirds. Significantly superior and excellent results observed in the cervical and middle thirds in the aloe vera subgroups in the current study support its optimal effect on PBS.

In the current study, the PBS generally decreased from the coronal towards the apical third. Evidence shows that the bond strength of fiber post to radicular dentin may vary along the root canal, due to reduction in dentin density and diameter from the coronal towards the apical region, no direct vision in deeper parts of the canal, difficult moisture control in the apical third, difficult flow and access of adhesive to the apical third, and reduction in degree of conversion of resin monomers, making the apical third the least desired part of the root for hybridization purposes (Aghdam et al. 2024). Similar results were reported in previous studies (Aghdam et al. 2024; Yu et al. 2022). Nonetheless, the present study found no significant difference in PBS among EGCG and control subgroups with/without using Endo‐Activator. Jouhar (2021) reported that type of cement can affect the efficacy of sonic irrigation technique, such that they found no significant difference in PBS between sonic and conventional irrigation techniques when using Panavia SA; while, the difference was significant when using Rely X U200. Only one type of cement was used in the present study to eliminate its confounding effect on the results.

Aloe vera, as a natural substance, showed superior results in MMP inhibition compared to EGCG after 6 months in the present study, which may indicate its superior inhibitory effects on MMPs. Although evidence shows that MMP‐2 and MMP‐12 are influenced by EGCG, the inhibitory effect of EGCG on MMP‐9 has also been confirmed in dentin (Vidal et al. 2014); whereas, aloe vera has inhibitory effects on MMP‐2 and MMP‐9 (Kudalkar et al. 2014). According to (Yu et al. 2022) MMP‐2 and MMP‐8 are extensively distributed in radicular dentin, and MMP‐2 (compared to MMP‐3 and MMP‐8) has the highest concentration in the apical third, where a higher bond strength is important to achieve (Yu et al. 2022). Thus, superior results yielded by aloe vera appear to be due to its greater inhibitory effects on MMPs available in radicular dentin. (Dax and Abraham 2020) demonstrated the highest PBS in glass fiber posts cemented with LuxaCore Z Dual core build‐up cement. Thus, a light transmitting fiber post, which is a type of glass fiber post (Moazami and Alaghehmand 2007), was used and cemented with LuxaCore Z Dual in the current study. However, due to the applied mechanical and chemical radicular dentin treatments in the present study, the range of obtained PBS values was much higher than that in the study by (Dax and Abraham 2020).

According to the present results, it can be concluded that active washing of the post space and simultaneous use of an effective natural MMP inhibitor such as aloe vera can increase the PBS, improve the durability of restorative treatments, and subsequently reduce the patient's treatment costs in the long‐term.

This study had some limitations. Only one type of activation system was tested. Additional studies are needed to assess the impact of alternative irrigation systems or protocols. Functional loads applied to the teeth in dental arch are variable and can affect the bond strength, which were absent in the present study, and should be addressed in future investigations. Future studies are also recommended to use fatigue loading for assessment of PBS of fiber posts. Also, other natural inhibitors, such as chlorhexidine, alcohol, and sodium fluoride (Alaghemand et al. 2014, 2013; Alaghehmad et al. 2018; Samani et al. 2018) should be evaluated and compared with aloe vera to find the most effective MMP inhibitors for use in the root canal system. Furthermore, the effects of aging by thermocycling were not evaluated in the present study, and should be taken into account in future studies. It is also suggested to formulate natural MMP inhibitors in combination with bonding agent primers. Additionally, future studies are recommended to use a combination of EGCG and aloe vera in bonding agents and assess their interaction effect on PBS.

## 5 Conclusion

Irrigation with Endo‐Activator along with the application of EGCG and aloe vera improved the PBS of fiber post to radicular dentin and its durability; this effect was greater in the cervical third in the aloe vera subgroup both immediately and after 6 months. However, when EGCG and aloe vera were used alone without Endo‐Activator, the increase in PBS was only significant in the aloe vera subgroup.

## Author Contributions

**Nadia Elyassi Gorji:** conceptualization, methodology, investigation, and reviewing the paper. **Homayoun Alaghemand:** conceptualization, project administration, and reviewing the paper. **Faraneh Mokhtarpour:** super‐vision and data analysis. **Elham Mahmoudnia:** super‐vision and methodology.

## Consent

The authors have nothing to report.

## Conflicts of Interest

The authors declare no conflicts of interest.

## Data Availability

The data that support the findings of this study are available from the corresponding author upon reasonable request.

## References

[cre270255-bib-0001] Aghdam, M. K. , M. Bahari , N. Mohammadi , et al. 2024. “Push‐Out Bond Strength of Fiber Posts to Overflared Root Canals in Different Root Regions: Effect of Reinforcement Techniques.” Frontiers in Dentistry 21: 24. 39104788 10.18502/fid.v21i24.16115 PMC11298702

[cre270255-bib-0002] Alaghehmad, H. , E. Mansouri , B. Esmaili , A. Bijani , S. Nejadkarimi , and M. Rahchamani . 2018. “Effect of 0.12% Chlorhexidine and Zinc Nanoparticles on the Microshear Bond Strength of Dentin With a Fifth‐Generation Adhesive.” European Journal of Dentistry 12, no. 1: 105–110. 29657533 10.4103/ejd.ejd_172_16 PMC5883460

[cre270255-bib-0003] Alaghemand, H. , B. Esmaeili , P. Firouz , et al. 2014. “A Comparative Study of the Preventive Effect of Chlorhexidine 0.12% and Nano Zinc Oxide Particles on the Distraction of Collagen Scaffolding of the Hybrid Layer by Two Immunohistochemistry and Microleakage Tests.” Dentistry and Medical Research 2, no. 2: 33–38.

[cre270255-bib-0004] Alaghemand, H. , M. Mirzae , E. Ahmadi , and A. Saidi . 2013. “Effect of Different Post‐Space Pretreatments on Fiber Post Bonding to Root Dentine.” Dental Research Journal 10, no. 4: 545–552. 24130594 PMC3793422

[cre270255-bib-0006] Aziz, T. T. , and I. M. Al Zaka . 2019. “Efficacy of XP‐Endo Finisher, XP‐Endo Finisher R, CanalBrush and EndoActivator in the Removal of Intracanal Medicament (an In Vitro Study).” International Journal of Scientific Research 8, no. 1: 919–928.

[cre270255-bib-0007] Bengoa, F. P. , M. C. Arze , C. M. Noguera , et al. 2020. “Effect of Ultrasonic Cleaning on the Bond Strength of Fiber Posts in Oval Canals Filled With a Premixed Bioceramic Root Canal Sealer.” Restorative Dentistry & Endodontics 45, no. 2: e19. 32483536 10.5395/rde.2020.45.e19 PMC7239684

[cre270255-bib-0008] Bhandari, S. , R. Kondody , A. Nair , R. Mathew , K. P. Divakar , and M. Nambiar . 2021. “Evaluation of Aloe vera as Matrix Metalloproteinase Inhibitor in Human Dentin With and Without Dentin‐Bonding Agent: An: In Vitro: Study.” Journal of Conservative Dentistry 24, no. 5: 491–495. 35399770 10.4103/jcd.jcd_474_21 PMC8989161

[cre270255-bib-0009] Bitter, K. , K. Priehn , P. Martus , and A. M. Kielbassa . 2006. “In Vitro Evaluation of Push‐Out Bond Strengths of Various Luting Agents to Tooth‐Colored Posts.” Journal of Prosthetic Dentistry 95, no. 4: 302–310. 16616128 10.1016/j.prosdent.2006.02.012

[cre270255-bib-0010] Bolles, J. A. , J. He , K. K. H. Svoboda , E. Schneiderman , and G. N. Glickman . 2013. “Comparison of Vibringe, EndoActivator, and Needle Irrigation on Sealer Penetration in Extracted Human Teeth.” Journal of Endodontics 39, no. 5: 708–711. 23611397 10.1016/j.joen.2013.01.006

[cre270255-bib-0011] Breschi, L. , T. Maravic , S. R. Cunha , et al. 2018. “Dentin Bonding Systems: From Dentin Collagen Structure to Bond Preservation and Clinical Applications.” Dental Materials 34, no. 1: 78–96. 29179971 10.1016/j.dental.2017.11.005

[cre270255-bib-0012] Bryce, G. , N. MacBeth , K. Gulabivala , and Y. L. Ng . 2018. “The Efficacy of Supplementary Sonic Irrigation Using the EndoActivator® System Determined by Removal of a Collagen Film From an Ex Vivo Model.” International Endodontic Journal 51, no. 4: 489–497. 29106737 10.1111/iej.12870

[cre270255-bib-0013] Dax, S. , and D. Abraham . 2020. “Need for an Alternative Method to Cement Fiber‐Reinforced Posts—A Pushout Bond Strength Analysis.” Journal of Conservative Dentistry 23, no. 3: 240–243. 33551592 10.4103/JCD.JCD_345_20 PMC7861071

[cre270255-bib-0014] Du, X. , X. Huang , C. Huang , Y. Wang , and Y. Zhang . 2012. “Epigallocatechin‐3‐Gallate (EGCG) Enhances the Therapeutic Activity of a Dental Adhesive.” Journal of Dentistry 40, no. 6: 485–492. 22421091 10.1016/j.jdent.2012.02.013

[cre270255-bib-0015] Garcia‐Contreras, R. , P. A. Chavez‐Granados , C. A. Jurado , B. Aranda‐Herrera , K. I. Afrashtehfar , and H. Nurrohman . 2023. “Natural Bioactive Epigallocatechin‐Gallate Promote Bond Strength and Differentiation of Odontoblast‐Like Cells.” Biomimetics 8, no. 1: 75. 36810406 10.3390/biomimetics8010075 PMC9944806

[cre270255-bib-0016] Ghazvehi, K. , A. Saffarpour , and S. Habibzadeh . 2022. “Effect of Pretreatment With Matrix Metalloproteinase Inhibitors on the Durability of Bond Strength of Fiber Posts to Radicular Dentin.” Clinical and Experimental Dental Research 8, no. 4: 893–899. 35726182 10.1002/cre2.569 PMC9382050

[cre270255-bib-0017] de Gregorio, C. , R. Estevez , R. Cisneros , C. Heilborn , and N. Cohenca . 2009. “Effect of EDTA, Sonic, and Ultrasonic Activation on the Penetration of Sodium Hypochlorite Into Simulated Lateral Canals: An In Vitro Study.” Journal of Endodontics 35, no. 6: 891–895. 19482193 10.1016/j.joen.2009.03.015

[cre270255-bib-0018] Hass, V. , I. V. Luque‐Martinez , M. F. Gutierrez , et al. 2016. “Collagen Cross‐Linkers on Dentin Bonding: Stability of the Adhesive Interfaces, Degree of Conversion of the Adhesive, Cytotoxicity and In Situ MMP Inhibition.” Dental Materials 32, no. 6: 732–741. 27087688 10.1016/j.dental.2016.03.008

[cre270255-bib-0019] Ismiyatin, K. , S. Goenharto , W. Irsya , P. Tanjung Sari , O. V. Widjaja , and R. P. Sari . 2022. “Potential of Epigallocatechin‐3‐Gallate as Chelating Agent Against Matrix Metalloproteinase Expression and as Cross‐Linking Agent Towards Hybrid Layer in Dentin Collagen: A Review.” Malaysian Journal of Medicine and Health Sciences 18, no. S6: S71–S77.

[cre270255-bib-0020] Jouhar, R. 2021. “Effect of Sonic Activation on Push‐Out Bond Strength of Fiber Post: An In Vitro Study.” Materials 14, no. 17: 5038. 34501127 10.3390/ma14175038 PMC8433804

[cre270255-bib-0021] Kudalkar, M. , A. Nayak , K. Bhat , and R. Nayak . 2014. “Effect of *Azadirachta indica* (Neem) and Aloe Vera as Compared to Subantimicrobial Dose Doxycycline on Matrix Metalloproteinases (MMP)‐2 and MMP‐9: An: In‐Vitro: Study.” AYU: An International Quarterly Journal of Research in Ayurveda 35, no. 1: 85–89. 10.4103/0974-8520.141947 PMC4213975 25364206

[cre270255-bib-0022] de Macedo, F. A. A. , N. O. Souza , M. V. S. Lemos , D. M. De‐Paula , S. L. Santiago , and V. P. Feitosa . 2019. “Dentin Bonding and Physicochemical Properties of Adhesives Incorporated With Epigallocatechin‐3‐Gallate.” Odontology 107, no. 1: 23–28. 29796959 10.1007/s10266-018-0367-0

[cre270255-bib-0023] Mancini, M. , L. Cerroni , L. Iorio , E. Armellin , G. Conte , and L. Cianconi . 2013. “Smear Layer Removal and Canal Cleanliness Using Different Irrigation Systems (EndoActivator, EndoVac, and Passive Ultrasonic Irrigation): Field Emission Scanning Electron Microscopic Evaluation in an In Vitro Study.” Journal of Endodontics 39, no. 11: 1456–1460. 24139274 10.1016/j.joen.2013.07.028

[cre270255-bib-0024] Mazzoni, A. , V. Angeloni , A. Comba , et al. 2018. “Cross‐Linking Effect on Dentin Bond Strength and MMPs Activity.” Dental Materials 34, no. 2: 288–295. 29179972 10.1016/j.dental.2017.11.009

[cre270255-bib-0025] Messias, D. C. , M. F. B. da Silva , N. S. de Faria , T. R. Dias‐Arnez , F. J. Rached‐Júnior , and A. Sousa . 2021. “Effect of Epigallocatechin‐3‐Gallate and Thermal Cycling on the Bond Strength of Resin Cements to the Root Dentin.” Odontology 109, no. 4: 854–859. 33963943 10.1007/s10266-021-00610-7

[cre270255-bib-0026] Moazami, S. , and H. Alaghehmand . 2007. “Effect of Light Conducting Cylindrical Inserts on Gingival Microleakage.” Journal of Dentistry 4, no. 1: 32–36.

[cre270255-bib-0028] Moustafa, N. M. , R. Afifi , and M. A. Niazy . 2020. “Comparative Evaluation for the Effect of Green Tea, Aloe Vera and Chlorhexidine on Dentin Erosion.” Future Dental Journal 1, no. 1: 1–8.

[cre270255-bib-0029] de Paris Matos, T. , J. Perdigão , E. De Paula , et al. 2020. “Five‐Year Clinical Evaluation of a Universal Adhesive: A Randomized Double‐Blind Trial.” Dental Materials 36, no. 11: 1474–1485. 32933775 10.1016/j.dental.2020.08.007

[cre270255-bib-0030] Pheenithicharoenkul, S. , and A. Panichuttra . 2016. “Epigallocatechin‐3‐Gallate Increased the Push Out Bond Strength of an Epoxy Resin Sealer to Root Dentin.” Dental Materials Journal 35, no. 6: 888–892. 27680035 10.4012/dmj.2016-137

[cre270255-bib-0031] Ruddle, C. 2015. “Endodontic Disinfection: Tsunami Irrigation.” Saudi Endodontic Journal 5, no. 1: 1–12.

[cre270255-bib-0032] Samani, Y. , H. Alaghehmand , Z. Jafari , et al. 2018. “Sodium Fluoride Addition to a Two‐Step Etch‐and‐Rinse Adhesive System: Effect on Dentin Microtensile Bond Strength and Durability.” Caspian Journal of Dental Research 7, no. 2: 16–23.

[cre270255-bib-0033] Sorourhomayoun, S. , H. Alaghehmand , S. Mahjoub , S. Khafri , and M. Ghasempour . 2021. “Shear Bond Strength of Composite to Primary Enamel Teeth Treated With Different Concentrations and Various Molecular Weights of Chitosan.” Caspian Journal of Dental Research 10, no. 1: 35–41.

[cre270255-bib-0034] Thakur, P. , U. Kumar , and H. Singh . 2022. “Assessment of Effectiveness of Two Techniques in Removal of Calcium Hydroxide Medication From Root Canals—An In Vitro Study.” Journal of Advanced Medical and Dental Sciences Research 10, no. 1: 105–108.

[cre270255-bib-0035] Victorino, K. , M. Kuga , M. H. Duarte , B. Cavenago , M. R. Só , and J. Pereira . 2016. “The Effects of Chlorhexidine and Ethanol on Push‐Out Bond Strength of Fiber Posts.” Journal of Conservative Dentistry 19, no. 1: 96. 26957803 10.4103/0972-0707.173210 PMC4760025

[cre270255-bib-0036] Vidal, C. M. P. , T. R. Aguiar , R. Phansalkar , et al. 2014. “Galloyl Moieties Enhance the Dentin Biomodification Potential of Plant‐Derived Catechins.” Acta Biomaterialia 10, no. 7: 3288–3294. 24721612 10.1016/j.actbio.2014.03.036 PMC4041811

[cre270255-bib-0037] Yu, H. H. , J. Liu , Z. X. Liao , et al. 2022. “Location of MMPs in Human Radicular Dentin and the Effects of MMPs Inhibitor on the Bonding Stability of Fiber Posts to Radicular Dentin.” Journal of the Mechanical Behavior of Biomedical Materials 129: 105144. 35290854 10.1016/j.jmbbm.2022.105144

[cre270255-bib-0038] Zhao, S. , Y. Zhang , Y. Chen , X. Xing , Y. Wang , and G. Wu . 2023. “Evaluation of Chitosan‐Oleuropein Nanoparticles on the Durability of Dentin Bonding.” Drug Design, Development and Therapy 17: 167–180. 36712950 10.2147/DDDT.S390039 PMC9879028

